# Comparison of causative microorganisms of posttraumatic endophthalmitis with and without retained intraocular foreign bodies

**DOI:** 10.1186/s12886-021-02130-y

**Published:** 2021-10-25

**Authors:** Yao Yang, Feng Mei, Jiaqi Lin, Jingyu Liao, Kaili Wu, Fang Duan

**Affiliations:** Zhongshan Ophthalmic Center, State Key Laboratory of Ophthalmology, Sun Yat-sen University, 54 Xianlie Road, Guangzhou, 510060 China

**Keywords:** Open globe injury, Endophthalmitis, Intraocular foreign body, Pathogens

## Abstract

**Background:**

The goals of this work were to report the demographic characteristics of patients with clinically diagnosed endophthalmitis with or without intraocular foreign bodies (IOFBs) and to analyze the causative microorganisms.

**Methods:**

A retrospective analysis was conducted on 1257 patients with clinically diagnosed posttraumatic endophthalmitis who were admitted to Zhongshan Ophthalmic Center between January 1, 2013, and August 31, 2020.

**Results:**

Of the 1257 patients with clinically diagnosed posttraumatic endophthalmitis, 452 (36.0%) patients had IOFBs. Male dominance was more common among the patients with IOFBs than the patients without IOFBs. The average age of the patients with IOFBs was older than that of the patients without IOFBs. The most common microbial pathogens in these two groups were Gram-positive cocci and Gram-negative bacilli. Gram-positive bacilli were more common in the patients with IOFBs than in those without IOFBs (17.9 vs. 9.4%), and Bacillus spp. accounted for 12.6 and 5.5%, respectively. Fungi were less abundant in the patients with IOFBs than in those without IOFBs (8.0 vs. 15.6%).

**Conclusions:**

Patients with IOFBs were mostly male and older than those without IOFBs. Gram-positive bacilli were more common and fungi were less common in patients with IOFBs than in those without IOFBs.

## Background

Posttraumatic endophthalmitis is a potentially devastating complication of open globe injuries and is of particular concern due to its tendency toward rapid vision loss and even blindness. Retained intraocular foreign bodies (IOFBs) occur in 18–41% of ocular trauma cases [1] and escalate the development of endophthalmitis. The prevalence of posttraumatic endophthalmitis has been reported to vary widely, i.e., from 0.9 to 11.9% [2–5], and may be much higher, i.e., from 6.9 to 30%, in cases of intraocular foreign body (IOFB) injury [6–8]. In addition, IOFBs are reported to be present in 43% of eyes diagnosed with traumatic endophthalmitis [9].

The spectrum of causative organisms of posttraumatic endophthalmitis varies in different regions. Most of the previous studies have reported that bacteria account for approximately 80–90% of culture-positive cases, and Gram-positive cocci are the most common isolates, followed by Gram-negative bacilli and Gram-positive bacilli [10, 11]. IOFBs have been reported as risk factors in many studies. Several studies have reported high incidence of Bacillus species endophthalmitis following open globe injuries, particularly in the setting of an IOFB or soil contamination [11–14]. However, the causative microorganisms of posttraumatic endophthalmitis with and without retained IOFBs are less well known.

Our previous study reported that the locations and types of IOFBs were related to the development of endophthalmitis [15]. Therefore, it is necessary to determine whether the presence of intraocular foreign bodies in patients with traumatic endophthalmitis has any effect on the distribution of pathogenic microorganisms in endophthalmitis. Thus, the purposes of the current study were to review the demographic characteristics of patients with clinically diagnosed endophthalmitis and to analyze the causative microorganisms with or without IOFBs. These findings will provide a reference for guiding the treatment of endophthalmitis.

## Methods

### Population

A retrospective review was conducted of patients clinically diagnosed with endophthalmitis with open globe injuries at the Zhongshan Ophthalmic Center (ZOC), Sun Yat-sen University (Guangzhou, China) from January 1, 2013, to August 31, 2020. This study was performed in compliance with the principles of the Declaration of Helsinki and was approved by the Institutional Ethics Committee of Zhongshan Ophthalmic Center, Sun Yat-sen University. In our study, clinical endophthalmitis was defined as any deterioration in clinical signs, such as hypopyon, decline in visual acuity, purulent discharge and positive microbial culture. The data in the case report format revealed no linkage to the patient identities, and the patient data confidentiality was protected. Data on demographic characteristics, including patient age and sex, medical records and laboratory results, were collected and analyzed. The type of IOFB was recorded as the name of the pathogenic object and classified as metallic or nonmetallic. The longest diameter of each IOFB was used to categorize the IOFBs into 4 size groups: <3 mm, 3–5 mm, >5–10 mm, and >10 mm.

### Pathogen isolation and identification

Pathogen cultures were undertaken using patients’ aqueous humor or vitreous or eye contents. The aqueous humor was aspirated from the anterior chamber through the limbus with a needle using a 1-mL syringe. Vitreous specimens were collected through the pars plana. Eye contents removed during enucleation were collected. The specimens were inoculated in nutrient broth overnight at 37 °C. Subsequently, the broth was inoculated onto sheep blood agar and potato glucose agar for the growth of bacterial and fungal cultures, respectively. Bacterial isolates were identified using an automated system (VITEK 2 compact BioMérieux, Inc., Marcy l’Étoile, France). Fungal isolates were identified by experienced technicians according to fungal morphology.

### Statistical analysis

All analyses were performed using SPSS version 16.0 (SPSS Inc., Chicago, IL, USA). The characteristics of the study population and the culture results were summarized using means and standard deviations for continuous variables and percentages for categorical variables. Differences were considered to be significant at *P* < 0.05.

## Results

In the current study, a total of 1257 patients were clinically diagnosed with endophthalmitis with open globe injuries; among them, 1033 patients were cultured for bacteria and fungi, and 383 patients yielded positive cultures, with a positive culture rate of 37.1% (383/1033). The positive culture rate for the patients with IOFBs was 38.0% (146/384), while that in the patients without IOFBs was 36.5% (237/649). There was no significant difference between these two groups (*P* = 0.629). Of the 1257 patients with clinically diagnosed posttraumatic endophthalmitis, 452 (36.0%) patients had IOFBs; furthermore, of the 383 patients with culture-proven endophthalmitis, 146 (38.1%) had IOFBs. Of 452 patients with IOFBs, 376 had IOFBs removed in our hospital, including 6 patients with multiple foreign bodies, 3 with eyelashes and metallic bodies, and 3 with eyelashes and nonmetallic bodies. Therefore, a total of 382 IOFBs were removed, with 70.9% being metallic and 29.1% being nonmetallic bodies. The percentages of IOFBs within the <3 mm, 3–5 mm, >5–10 mm, and >10 mm groups were 32.7, 34.0, 19.1, and 14.1%, respectively.

As shown in Table 1, of the 1257 clinically diagnosed endophthalmitis patients, 1051 patients were male, and 206 were female. In addition, 452 patients had IOFBs, and 805 patients did not have IOFBs. The proportion of males was 89.8% among patients with IOFBs, which was significantly higher than the 80.1% proportion of males among patients without IOFBs (*P* < 0.001). The age of the patients with clinically diagnosed endophthalmitis with IOFBs ranged from 1 to 78 years, with a mean of 38.3±15.6 years, which surpassed the age of the patients without IOFBs, which ranged from 7 months to 78 years, with a mean of 27.9±20.5 (*P* < 0.001). Of the 383 patients with culture-proven endophthalmitis, the age of the patients with IOFBs ranged from 1 to 67 years, with a mean of 38.8±15.7 years, and the age of the patients without IOFBs ranged from 8 months to 78 years, with a mean of 31.7±20.8 years. The average age of the patients with IOFBs was older than that of the patients without IOFBs (*P* < 0.001). The proportions of males were 86.3% among the patients with IOFBs and 81.4% among the patients without IOFBs (*P* = 0.021).

**Table 1:** Comparison of the demographic information of endophthalmitis with or without intraocular foreign bodies

	Clinically diagnosed endophthalmitis (*n*=1257)		*P* value	Culture-proven endophthalmitis (*n*=383)		*P* value
	With IOFBs	Without IOFBs		With IOFBs	Without IOFBs	
Sex			*P* < 0.001			*P* = 0.21
Male	406	645		126	193	
Female	46	160		20	44	
Age	38.3±15.6	27.9±20.5	*P* < 0.001	38.8±15.7	31.7±20.8	*P* < 0.001
Total	452	805		146	237	

**Fig. 1:**
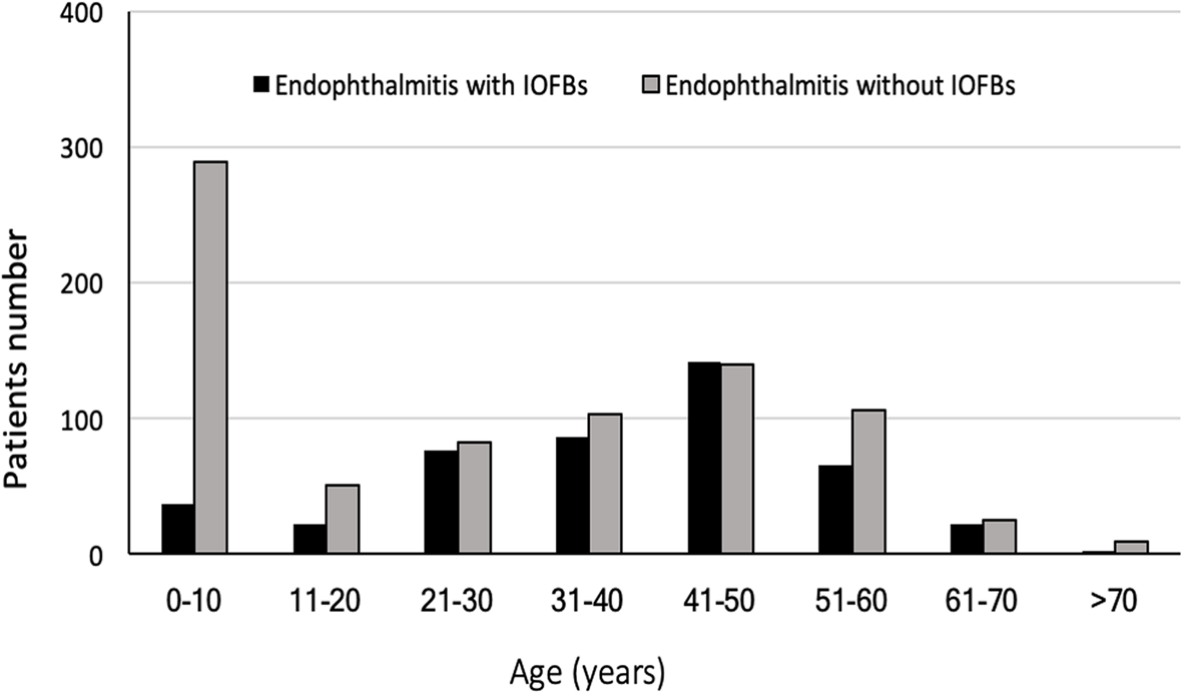
Demographics of 1257 patients with clinically diagnosed posttraumatic endophthalmitis

The age distributions of the 1257 patients with clinically diagnosed posttraumatic endophthalmitis are shown in Fig. 1. The distribution of children aged between 0 and 10 was the most concentrated, followed by middle-aged patients between 41 and 50 years old. In the range of 11–50 years old, the prevalence increased with age, and the number of patients older than 60 years of age decreased sharply. Although the number of patients with IOFBs in the 41–50 age group was slightly greater than that without IOFBs, the numbers of patients without IOFBs in the other age groups were greater than those with IOFBs.

The distribution of 407 isolates of 383 patients is shown in Table 2. Overall, bacteria were more predominant pathogens than fungi. In detail, the most common causative pathogens were Gram-positive cocci (45.0 and 52.7% in the groups with IOFBs and without IOFBs, respectively), followed by Gram-negative bacilli (27.2 and 20.7% in the groups with IOFBs and without IOFBs, respectively). Gram-positive bacilli were more common in the patients with IOFBs than in those without IOFBs (17.9 vs. 9.4%), accounting for 12.6 and 5.5%, respectively. In detail, Bacillus cereus was the most common gram-positive bacilli and accounted for 10.6% in the patients with IOFBs and 4.3% in the patients without IOFBs. Fungi were less abundant in the patients with IOFBs than in those without IOFBs (8.0 vs. 15.6%). In detail, there were 4 (2.7%) Aspergillus spp. isolated from the patients with IOFBs, and 14 (5.5%) were isolated from the patients without IOFBs. The detailed distributions of other pathogens are detailed in Table 2.

**Table 2:** Comparison of the causative isolates of endophthalmitis with or without intraocular foreign bodies

	Endophthalmitis with IOFBs, N (%)	Endophthalmitis without IOFBs, N (%)	Total N (%)
**Gram-positive cocci**	**68 (45.0)**	**135 (52.7)**	**203 (49.9)**
Coagulase-negative Staphylococcus	52 (34.4)	74 (28.9)	126 (31.0)
Streptococcus	7 (4.6)	32 (12.5)	39 (9.6)
Enterococcus	4 (2.7)	9 (3.5)	13 (3.2)
Kocuria spp.	1 (0.7)	8 (3.1)	9 (2.2)
Granulicatella	3 (2.0)	1 (0.4)	4 (1.0)
Leuconostoc	-	4 (1.6)	4 (1.0)
*Staphylococcus aureus*	-	3 (1.2)	3 (0.7)
Others	1 (0.7)	4 (1.6)	4 (1.0)
**Gram-positive bacilli**	**27 (17.9)**	**24 (9.4)**	**51 (12.5)**
*Bacillus cereus*	16 (10.6)	11 (4.3)	27 (6.6)
*Bacillus subtilis*	3 (2.0)	3 (1.2)	6 (1.5)
*Propionibacterium acnes*	1 (0.7)	3 (1.2)	4 (1.0)
Others	7 (4.6)	7 (2.7)	14 (3.4)
**Gram-negative cocci**	**2 (1.3)**	**2 (0.8)**	**5 (1.2)**
Neisseria	2 (1.3)	2 (0.8)	4 (1.0)
**Gram-negative bacilli**	**41 (27.2)**	**53 (20.7)**	**94 (23.1)**
*Pseudomonas aeruginosa*	6 (4.0)	6 (2.3)	12 (2.9)
*Enterobacter cloacae*	3 (2.0)	7 (2.7)	10 (2.5)
Aeromonas	4 (2.7)	4 (1.6)	8 (2.0)
Klebsiella	1 (0.7)	1 (0.4)	2 (0.5)
Acinetobacter	5 (3.3)	1 (0.4)	6 (1.5)
Xanthomonas	2 (1.3)	4 (1.6)	6 (1.5)
Serratia	2 (1.3)	3 (1.2)	5 (1.2)
*Escherichia coli*	1 (0.7)	3 (1.2)	4 (1.0)
*Enteric bacilli*	2 (1.3)	3 (1.2)	5 (1.2)
Sphingomonas	3 (2.0)	1 (0.4)	4 (1.0)
Pantoea	1 (0.7)	2 (0.8)	3 (0.7)
Citrobacter	3 (2.0)	-	3 (0.7)
Others	8 (5.3)	18 (7.0)	26 (6.3)
**Fungus**	**12 (8.0)**	**40 (15.6)**	**52 (12.8)**
Aspergillus spp.	4 (2.7)	14 (5.5)	18 (4.4)
Candida	1 (0.7)	4 (1.6)	5 (1.2)
Fusarium spp.	4 (2.7)	7 (2.7)	11 (2.7)
Mucor	-	4 (1.6)	4 (1.0)
Penicillium	-	1 (0.4)	1 (0.2)
Others	3 (2.0)	10 (3.9)	13 (3.2)
**Actinomycetes**	1 (0.7)	2 (0.8)	3 (0.7)
**Total**	**151 (100)**	**256 (100)**	**407 (100)**

There were 21 patients with mixed infections, and the majority of them had double bacterial infections. Among them, there were 5 cases with IOFBs and 16 cases without IOFBs. Simultaneous infection with three pathogens only occurred in patients without IOFBs. Detailed information on the 21 patients is shown in Table 3.

**Table 3:** Mixed infections of 21 patients with posttraumatic endophthalmitis

Patients	IOFBs	Bacteria	Fungus
1	Yes	*Staphylococcus saprophyticus*	Fusarium
2	Yes	*Enterobacter cloacae*	Mucor
3	Yes	*Bacillus cereus/Pseudomonas aeruginosa*	-
4	Yes	*Pseudomonas aeruginosa/Micrococcus luteus*	-
5	Yes	*Streptococcus anginosus/Staphylococcus epidermidis*	-
6	No	*Bacillus cereus/Kocuria roseus*	-
7	No	*Bacillus megaterium/Streptococcus gordonii*	-
8	No	*Pantoea agglomerans/Staphylococcus epidermidis*	-
9	No	*Propionibacterium bullosum/Escherichia coli*	-
10	No	*Enterobacter albopictus/Streptococcus salivarius*	-
11	No	*Staphylococcus epidermidis/Aeromonas sobria*	-
12	No	*Staphylococcus epidermidis/Staphylococcus saprophyticus*	-
13	No	*Staphylococcus epidermidis/Staphylococcus saprophyticus*	-
14	No	*Bacillus cereus*	*Aspergillus flavus*
15	No	*Staphylococcus epidermidis/Staphylococcus aureus*	-
16	No	*Bacillus cereus/Abiotrophia defectiva*	-
17	No	*Enterococcus gallinarum/Enterobacter albopictus*	-
18	No	*Chryseobacterium indologenes/Delftia acidovorans*	-
19	No	*Brevundimonas diminuta/Staphylococcus epidermidis*	*Aspergillus flavus*
20	No	*Staphylococcus epidermidis/Streptococcus mutans/streptococcus oralis*	-
21	No	*Staphylococcus epidermidis/Staphylococcus aureus/Mycobacterium*	-

## Discussion

Posttraumatic endophthalmitis remains an important complication of open globe injuries, and IOFBs are a risk factor for the development of endophthalmitis. In this study, our goal was to compare the demographic characteristics and causative pathogens of endophthalmitis with or without IOFBs. We found that of 1257 patients with clinically diagnosed posttraumatic endophthalmitis, 452 (36.0%) patients had IOFBs. Males dominated these two groups, and males were more common among the patients with IOFBs than among the patients without IOFBs. The average age of the patients with IOFBs was older than that of the patients without IOFBs. The most common microbial pathogens in these two groups were Gram-positive cocci and Gram-negative bacilli. Gram-positive bacilli were more common in the patients with IOFBs than in those without IOFBs (17.9 vs. 9.4%), and Bacillus spp. accounted for 12.6 and 5.5%, respectively. Fungi were less abundant in the patients with IOFBs than in those without IOFBs (8.0 vs. 15.6%).

In the current study, among the 1257 patients with clinically diagnosed endophthalmitis and the 383 with culture-positive endophthalmitis, the prevalence rates of IOFBs were 36.0 and 38.1%, respectively, which were slightly lower than the 43% reported in a study of 67 culture-positive posttraumatic endophthalmitis patients [9]. Additionally, we found that endophthalmitis occurred predominantly in men (83.6%), and the mean age of all the patients was 38.3 ±15.6 years old. The mean age of the patients (38.3 ± 15.6) with IOFBs was significantly older than that of the patients (27.9 ± 20.5) without IOFBs. This finding might be due to sex-based behavior and male involvement in higher-risk working activities. In addition, only in the 41- to 50-year-old group were the patients close in number. In other age groups, the number of people without IOFBs was greater than that with IOFBs, especially in the age group under 10 years old. The great difference in the distribution of children under 10 years old between the two groups might explain why the average age of the patients with IOFBs was older than the age of those without IOFBs. A previous study in Southwest China reported that working-age males accounted for 79.1% of patients with IOFBs, and there were significant differences in age distributions [16], which is consistent with our results. Most previous studies of IOFBs have revealed that males predominate, and the average ages have been close to those in other findings. For example, Ratanapakorn et al. reported that males accounted for 93.3% of patients and that the mean age was 36.4 years for 359 consecutive patients with retained IOFBs in Thailand [17]. Anguita et al. reported that among 61 Latin American patients with IOFBs, males accounted for 97%, and the mean age was 37.9 years old [18]. In England, Bourke et al. reported that 23 patients with IOFBs were all male, with a mean age of 37.4 years old [19].

The isolation of microbial pathogens is an important step in clinical practice. In the current study, Gram-positive cocci were found in 203 eyes (49.9%), Gram-negative bacilli were found in 94 eyes (23.1%), and Gram-positive bacilli were found in 51 eyes (12.5%). Coagulase-negative Staphylococcus was the major causative pathogen, which is consistent with previous studies [9, 11–14, 20]. Gram-positive bacilli were more common in the patients with IOFBs than in those without IOFBs (17.9 vs. 9.4%). Bacillus spp. is a group of uncommon but aggressive pathogens and accounts for approximately 1.5–10.7% of infectious endophthalmitis cases [11–14]. Furthermore, Bacillus spp. was more common in cases of endophthalmitis following open globe injuries than in cases of postoperative and endogenous endophthalmitis [21]. For example, Jindal reported that Bacillus spp. accounted for 17.1% of posttraumatic endophthalmitis cases [22], which was similar to our results. In addition, we found that Bacillus spp. were more common in the patients with IOFBs than in those without IOFBs (12.6 vs. 5.5%). It has been reported that patients with traumatic ocular injuries have a high probability of being infected with *Bacillus cereus* if the injury is associated with a retained IOFB [23], which is consistent with our results. Fungal endophthalmitis following open-globe injuries is less common than bacterial endophthalmitis. In our series, fungi accounted for 8.0 and 15.6% in the patients with IOFBs and without IOFBs, respectively. The reasons for this difference still need to be further explored. We speculated that this might be related to the causes of ocular trauma and types of IOFBs. It has been reported that the most common agents in previous studies were Aspergillus, Candida and Fusarium [13, 14, 24, 25], which is consistent with our results. After comparison, we found that the distributions of most pathogens containing intraocular foreign bodies were similar, but there were different distributions of Bacillus species and fungi. In the current study, multiple infections accounted for 5.5% (21/383) of cases, with 1.3% (5/383) detected in the patients with IOFBs and 4.2% (16/383) in the patients without IOFBs. A previous study of 1593 patients with infectious endophthalmitis (82.6% with posttraumatic endophthalmitis) reported that 5.3% were infected by multiple pathogens [26], which was similar to our results.

The limitations of this study included its retrospective nature and the lack of visual acuity prognosis. Nevertheless, our study provides valid data for understanding the demographic characteristics and isolation of causative organisms from patients with or without IOFBs.

## Conclusions

In this study, our results demonstrated that 36.0% of patients had IOFBs among cases of posttraumatic endophthalmitis. Male dominance was more common among patients with IOFBs than among patients without IOFBs. The average age of the patients with IOFBs exceeded that of the patients without IOFBs. The most common microbial pathogens were Gram-positive cocci and Gram-negative bacilli in these two groups. Gram-positive bacilli were more common in the patients with IOFBs than in those without IOFBs. Fungi were less abundant in the patients with IOFBs than in those without IOFBs. Our findings demonstrated the different distributions of age, sex and microbial pathogens between patients with IOFBs and those without IOFBs.

## Data Availability

All the data used to support the findings of this study are included within the article and are available from the corresponding author upon reasonable request.
